# Physical Activity and Psychological Symptoms in University Teachers Improved Post-COVID-19 Lockdown, but Sedentary Behavior Persisted

**DOI:** 10.3390/healthcare13151772

**Published:** 2025-07-22

**Authors:** Laura M. Navarro-Flores, Brajan J. Vivas-Sánchez, Jose María De La Roca-Chiapas, Victor K. Rodrigues Matsudo, Maciste H. Macias, Katya Vargas-Ortiz

**Affiliations:** 1 Department of Medical Sciences, University of Guanajuato, Leon 37000, Guanajuato, Mexico; lm.navarroflores@ugto.mx (L.M.N.-F.); brajansanchez@gmail.com (B.J.V.-S.);; 2 Department of Psychology, University of Guanajuato, Leon 37670, Guanajuato, Mexico; joseroca@ugto.mx; 3 Center of Studies of the Physical Fitness Research Laboratory, São Caetano do Sul 09520-320, São Paulo, Brazil; matsudo.celafiscs@gmail.com

**Keywords:** accelerometer, lockdown, mental health, movement patterns, sedentary behavior

## Abstract

**Background/Objectives**: This study aimed to determine whether the movement patterns and mental health of university teachers changed after returning to on-site class activities following the COVID-19 lockdown. Specifically, it compared levels of physical activity (PA), sedentary behavior time (SBT), active breaks (ABs), and symptoms of depression, anxiety, and stress among university teachers during online and on-site teaching periods. We also analyzed the association between movement patterns with psychological and anthropometric variables. **Methods**: University teachers who engaged in online teaching activities because of the COVID-19 restrictions and returned to on-site classes were included. Each teacher wore an accelerometer and answered the Depression Anxiety Stress Scales. The following parameters were assessed: SBT, light (LPA), moderate (MPA), and vigorous (VPA) (min/day); moderate–vigorous PA (MVPA) (min/week); steps/day and ABs/day. **Results**: Thirty-seven teachers with complete data from both phases were included. Once the on-site teaching activities resumed, LPA (9 min/day), MPA (6 min/day), total PA (20 min/day), MVPA (49 min/week), and steps/day (1100) significantly increased. While SBT showed no changes, ABs/day bouts increased. Depression and stress symptoms improved upon returning to on-site teaching activities. A positive association was identified between SBT and waist circumference (WC). There were negative associations between steps/day and MVPA with body mass index (BMI), steps/day with WC, and LPA with stress symptoms. **Conclusions**: Upon returning to on-site teaching activities, PA levels, steps/day, and ABs/day bouts all increased, although SBT remained elevated compared with during the lockdown. The teachers’ psychological symptoms improved. PA was associated with better health markers, while SBT was associated with increased WC.

## 1. Introduction

The onset and spreading of the coronavirus disease 2019 (COVID-19) were dealt with using rigorous confinement policies that significantly restricted people’s lifestyles [1]. Some studies were conducted during the first months of the pandemic using self-report questionnaires to measure defined outcomes. These showed decreased physical activity (PA) and a subsequent increase in sedentary behavior time (SBT) [2,3,4]. Furthermore, a positive correlation was detected between PA and mental well-being [5]. Similarly, increased SBT during leisure time was positively associated with physical health deterioration [6].

PA and SBT were also accelerometrically measured. Such studies were focused on special populations (elderly hypertensive individuals, young athletes, frail elderly individuals, adults with heart failure, and post bariatric adults) and reduced sample sizes (10–35 individuals). The results show that PA and the number of steps decreased whereas SBT increased during the COVID-19 lockdown [7,8,9,10,11].

Active breaks (ABs) represent a strategy to counteract SBT by engaging in PA. A 3 min AB bout following 30 min of sedentary behavior may improve glucose, insulin, and triglyceride levels, thereby improving the metabolic risk markers [12,13]. However, ABs were not quantified during the lockdown period.

On the other hand, mood disorders were associated with COVID-19 mortality [14] and physical inactivity was associated with a greater risk of hospitalization, admission to the intensive care unit, and death [15]. While physical activity demonstrated a dose–response preventive association with adverse COVID-19 outcomes, this effect remained significant even after adjusting for clinical and demographic characteristics [16].

Right from the start, the COVID-19 pandemic posed new challenges in the academic field. Home office activities were considered an important strategy to reduce exposure to the virus and, consequently, to limit the risk of infection [17]. However, this behavior had a negative impact on occupational conditions, including the physical and mental health status of the employees [18].

To date, no information regarding the COVID-19 lockdown impact on movement patterns displayed by teachers is available and the few published results were obtained using surveys. Concerning mental health status, a study conducted on a university staff population indicated that approximately 30% of them exhibited moderate to extremely severe symptoms of depression, anxiety, and stress during the lockdown period. In particular, administrative employees and teachers exhibited higher scores that were considered of concern when compared with students [19].

In October 2021, COVID-19 lockdown restrictions were gradually reduced in several countries [20]. However, there are no studies conducted on teachers describing the changes of PA levels and SBT using objective assessments, and very few studies have analyzed the psychological symptoms of mental health either during lockdown or afterwards. Despite being key contributors to society, teachers are often overlooked in the design of public health interventions, highlighting the need to better understand their health-related behaviors.

The identification of movement patterns and changes of psychological symptoms post-lockdown will reveal the existing issues to establish potential preventive and therapeutic actions aimed at improving the physical and mental health status of teachers in the short- and long-term. We hypothesized that, after the COVID-19 lockdown, university teachers would show increased levels of PA and ABs, as well as reduced SBT and lower levels of depression, anxiety, and stress symptoms compared with during the lockdown period.

The objective of this study was to compare the PA level, SBT, and ABs as assessed objectively in addition to the depression, anxiety, and stress symptoms displayed by university teachers during the COVID-19 lockdown period and afterwards. Secondarily, we also evaluated the correlation of PA, SBT, and ABs with psychological and anthropometric variables.

## 2. Materials and Methods

### 2.1. Study Design

This was a longitudinal and comparative study. The protocol was in agreement with the Declaration of Helsinki of 1975 (revised in 2013) [21] and it was previously approved by the Research Ethics Committee of the University of Guanajuato (CIBIUG-P04-2021, CIBIUG-P66-2021). Before each phase of the study, every subject signed the informed consent form where data confidentiality and the possibility of a voluntary withdrawal were stated. The size of the sample was selected for convenience purposes.

### 2.2. Participants

All participants met the following eligibility criteria: full-time faculty members at the University of Guanajuato, aged 30 to 60 years, including non-pregnant women and men. For the first phase of the study, conducted under confinement conditions, all teachers worked online for at least 8 h a day. Once the lockdown was lifted (second phase of the study), they returned to on-site teaching activities. Those individuals with musculoskeletal injuries compromising their movements did not participate in this study. Exclusion criteria were as follows: acute musculoskeletal injury (e.g., muscle sprains or tears), as well as those injuries requiring a cast and/or crutches, or any condition that may affect the normal routine during the period where the subject will be using an accelerometer device (one week), or that the use of the latter was less than 4 days/week or/and less than 10 h/day or did not include at least 1 weekend day.

### 2.3. Procedures

The call to participate in the first phase of the study was made public through institutional emails, advertising on the news portal of the University of Guanajuato, and by direct verbal communication with the teachers. A virtual briefing meeting was held with all the potential participants to explain the procedures involved in the study. It was asked to each possible participant to provide information about their type of contract with the university, the number of hours they spend in online teaching activities, and if they currently have any medical condition or if they will consume prescription drugs during the week of the study.

**Figure 1:**
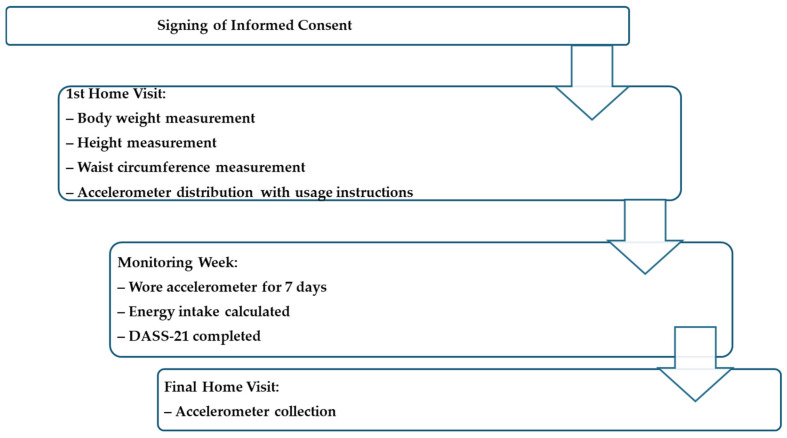
Flow chart showing the procedures.

The subjects that met the inclusion criteria received a home visit to measure their body weight (TANITA FitScan BC-730F, Tokyo, Japan) and height (SECA 213 portable stadiometer, Hamburg, Germany), as well as their WC (Lufkin W606PM, Sparks, NV, USA) up to 0.1 kg and 0.1 cm accuracies, respectively. All measurements and the identification of any technical measurement error were carried out based on the standardization established by the International Society for the Advancement of Kinanthropometry (Figure 1).

All participants were instructed to wear an accelerometer device on the right side of the waist for 7 consecutive days during the daytime but not during showering activities. Home visits were made for no more than 20 min and suitable sanitary measures were adopted to avoid COVID-19 transmission risks.

During the initial phase of the evaluation week, an illustrated brochure was provided to all subjects where the instructions to properly place and wear the accelerometer device were depicted. Additionally, a WhatsApp message was sent to them each morning as a reminder to wear their accelerometer device (Figure 1).

During the evaluation week, the energy consumption (kcal/day) was calculated using the Nutrikal software ^R^VO (Monterrey, Mexico) considering the data from a meal consumption registry that spanned three days (this included one day of the weekend). The meal consumption registries were established during online sessions. All participants answered a questionnaire in Google Forms regarding their health conditions, including questions from the Depression Anxiety Stress Scales 21 (DASS-21) questionnaire. Once the evaluation week concluded, they received a final home visit to collect the electronic device (Figure 1).

For the second phase of the study, emails (up to 3 attempts) or telephone calls (up to 3 attempts) were made to the teachers with complete data from the first phase. A virtual briefing meeting was held with those who agreed to participate to remind them the procedures involved in the study. Similar to the first phase, a brief home visit was made to collect weight, height, and WC measurements, and to hand over and initialize the accelerometer device. An illustrated brochure was provided to all subjects to remind them of the instructions to properly place and wear the accelerometer. Again, a WhatsApp message was sent to them each morning as a reminder to wear their accelerometer device.

For the second phase of the study, energy consumption was also evaluated and the subjects answered questions from the DASS-21 questionnaire. Once the second week of the evaluation had concluded, all teachers received a final home visit to collect the electronic device.

### 2.4. Objective Assessment of PA, SBT, and ABs

In both phases of the study all participants wore an ActiGraph accelerometer (wGT3X-BT, Pensacola, FL, USA). The device was programmed for a 60 Hz sample acceleration. The data was downloaded using the Actilife software version 6.13.4 in 60 s epochs. The accelerometer data recorded within a ≥ 600 min (10 h) period per day was considered valid, except for the periods where the device was not worn (non-wear condition) for at least 4 days of the week (three weekdays and one day of weekend) [22]. The non-wear periods were defined as 0 consecutive counts per minute (cpm) ≥ 90 min, and a ≥ 100 cpm tolerance up to 2 min. SBT, light PA (LPA), moderate PA (MPA), and vigorous PA (VPA) were measured as minutes/day, whereas moderate and vigorous PA (MVPA) was expressed in minutes/week to allow comparison with the PA global recommendations by the World Health Organization (WHO). The number of daily steps, SBT bout ≥ 30 min, and AB bouts ≥ 3 min after remaining in a sitting position for ≥ 30 min were also obtained. The PA level was defined using the cut-off points established by Freedson [23].

### 2.5. Description of Depression, Anxiety, and Stress Symptpms

The DASS-21 questionnaire was administered in both study phases. It had been previously validated in Spanish for use in the Mexican population, demonstrating a Cronbach’s alpha coefficient of 0.86 [24]. DASS-21 consists of 21 items based on a Likert scale of 4 response options (0–4 points). The scores concerning the items of special interest (depression, anxiety, and stress) were quantified. Symptom levels were assessed by considering specific cut-off point values as follows. For depression: mild 5–6, moderate 7–10, severe 11–13, and extremely severe 14 or above; for anxiety: mild 4, moderate 5–7, severe 8–9, and extremely severe 10 or above; for stress: mild 8–9, moderate 10–12, severe 13–16, and extremely severe 17 or above [25].

### 2.6. Statical Analysis

The Shapiro–Wilk test was used to establish the data distribution. Based on the latter, the data was expressed either as mean ± standard deviation, as the median value (min–max), or as percentages. To compare the changes between both phases, the Wilcoxon test was used for variables without a normal distribution, a paired t test was for used variables with normal distribution, and a Pearson chi-square was used to analyze the differences between proportions.

A Spearman or Pearson correlation analysis was performed to identify the relationship between movement patterns (LPA, MPA, VPA, MVPA, number of steps per day, SBT bouts ≥ 30 min per day, or AB bouts ≥ 3 min after ≥ 30 min of SBT) with either of the psychological parameters (depression, anxiety, and stress scores) and the anthropometric variables (BMI and WC). When a significant correlation was observed, a simple linear regression was used to assess the degree of association between the respective variables. A *p* < 0.05 value was considered significant.

## 3. Results

### 3.1. Participants

The data from the first phase was collected between June and October 2021 and that from the second phase was collected between April and November 2022. During the first phase, all academic activities were exclusively performed online, whereas those of the second phase were carried out during on-site teaching activities.

**Figure 2:**
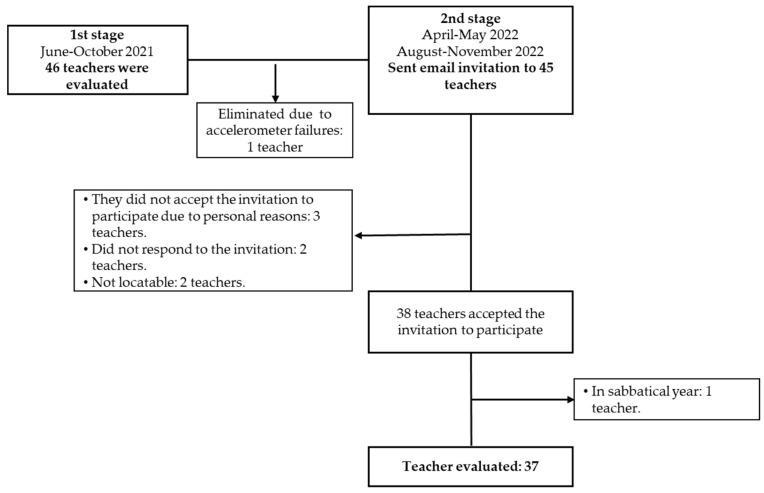
Flow chart showing the number of participating subjects.

During the first phase, all public spaces were open but with reduced capacity in order to avoid mass gatherings, and a proper distance among individuals was encouraged along with the use of face masks. Because of the public health dispositions implemented during such period, all data was collected when the subjects were at home as they continued their work activities online. Sixty-three teachers located in Guanajuato state were willing to participate; forty-six of them were eligible. The data from 45 subjects were included in first phase (Figure 2).

For the second phase of the study, the data was collected when teachers were engaged in on-site teaching activities, when most of the sanitary restrictions were released as 61% of the Mexican population had been immunized with at least two doses of the COVID-19 vaccine [26]. All 45 teachers with complete data from the first phase of the study were invited to participate. However, only 37 teachers were included in the final analysis because several declined participation, others could not be located, and some, after initially agreeing, took a sabbatical year (Figure 2).

In both phases of the study, the sample included a slightly higher number of male participants, with a mean age of 47.3 years. All teachers successfully completed the period where the accelerometer device had to be used daily. Additionally, a slightly higher number were overweight (Table 1). During the first phase of the study all subjects indicated that they worked online for 8 h/day or more.

For the second phase of the study, the WC parameter decreased significantly with a subsequent change in the BMI classification. The number of overweight teachers decreased and the those with normal weight concomitantly increased (Table 1).

### 3.2. Physical Activity Level, Sedentary Behavior Time, and Active Breaks

**Figure 3:**
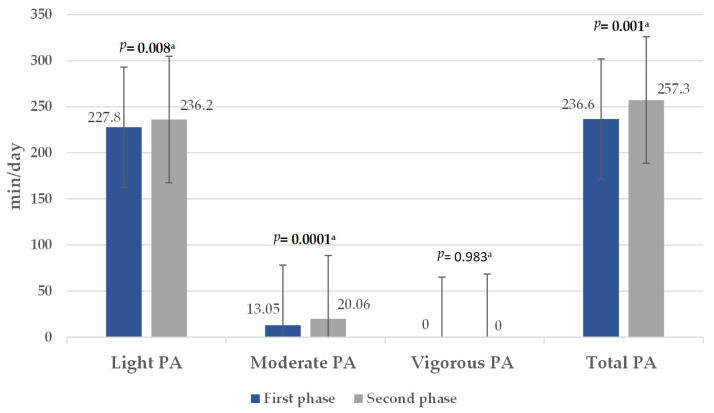
Comparison of the physical activity level in the first and second phases of the study. PA, physical activity, ^a^ Wilcoxon Test. Data expressed as median (min–max).

All teachers were mainly engaged in LPA during the first phase. Teachers accumulated 4909 (1526–12,583) steps/day. Once the COVID-19 lockdown was lifted, in the second phase of the study, the overall PA significantly increased mainly because LPA and MPA increased (Figure 3). Weekly MVPA also increased significantly from 101 (4–532) to 150 (14–589) min/week (*p* = 0.0001). Similarly, the number of steps per day increased from 4909 (1526–12,583) to 6107 (2348–15,555) (*p* = 0.0001).

An individual is classified as physically active when meeting the criteria established by the WHO Global Recommendations on Physical Activity for Health (performing at least 150 min/week MVPA). Thus, in the second phase of the study there was a significant increase in the number of physically active teachers from 27% in the first phase of the study to 51.4% in the second phase (*p* = 0.02).

**Figure 4:**
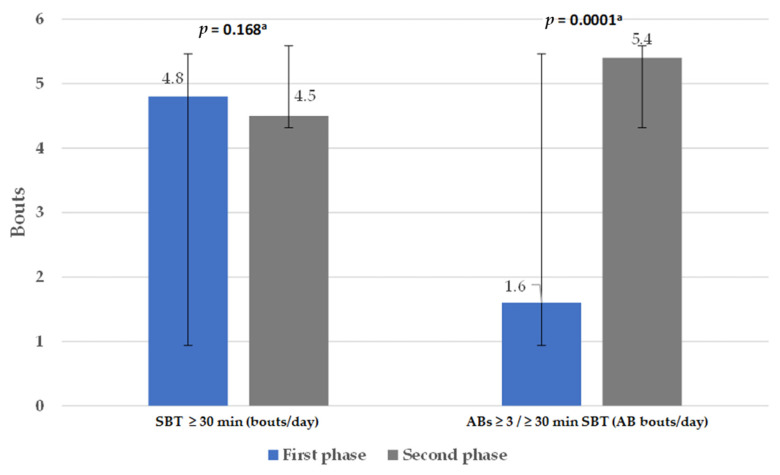
Comparison of the sedentary behavior time in the first and second phases of the study. ABs, active breaks; SBT, sedentary behavior time. ^a^ Paired-Samples *t* test. Data expressed as mean ± SD.

In the first phase of the study, the teachers spent 616.1 ± 89.9 min/day in SBT (70% of their daytime). For the second phase of the study the SBT decreased to 604.5 ± 113.3 min/day, however the difference was not significant (*p* = 0.390). The SBT and the SBT bouts ≥ 30 min/day periods showed no significant changes. However, the amount of AB bouts per day significantly increased (Figure 4).

### 3.3. Depression, Anxiety, and Stress Symptoms

During online teaching in the first phase of the study, 21.7% of the teachers exhibited depression, 16.7% anxiety, and 25% stress. Once the COVID-19 lockdown was lifted, the percentage of teachers with depression was 8.1%, whereas anxiety and stress symptoms decreased to 21.7%. A comparative analysis between the study phases showed a significant changed in the categories of depression, anxiety, and stress symptoms (Table 2). None of the participants took antidepressant medications.

### 3.4. Correlation Between Physical Activity Level, Sedentary Behavior Time, and Active Breaks with Mental Health Status and Body Measurements During the Second Phase of the Study

The daily parameters of number of steps, MPA, and VPA, as well as the weekly parameter MVPA negatively correlated with BMI. The number of steps per day and VPA negatively correlated with WC, whereas SBT per day positively correlated with the WC. The DASS-21 variables correlated positively with each other (Table 3).

Several simple linear regression models were evaluated. They showed that LPA, MVPA, and the number of steps were negatively associated with stress, BMI, and WC. Conversely, SBT was positively associated with WC (Table 4).

## 4. Discussion

This is the first study to compare PA levels, SBT, and psychological symptoms in university teachers during online and on-site teaching periods affected by COVID-19.

It was not possible to make a direct comparison with similar studies as only a few studies have assessed PA, SBT, and psychological aspects on teachers as subjects during and after the COVID-19 lockdown. However, some studies have considered similar aspects.

### 4.1. Physical Activity and Sedentary Behavior

During the second phase of the study, all teachers increased their LPA. Although there are no studies comparing the PA level of teachers during the pandemic and afterwards, the results were similar after comparing the changes before and during the lockdown. There are studies indicating that LPA, as evaluated by accelerometry [7] or by a questionnaire [27], was lower during the COVID-19 lockdown when compared with the conditions before these restrictive conditions.

Howard et al. (2015) [28] reported that either low-intensity LPA (such as cooking, ironing, or stretching) 60 min/day or high-intensity LPA (such as sweeping, gardening, and playing golf) 45 min/day are associated with 1 cm decreases of WC. Moreover, low- and high-intensity LPA were both associated with decreased levels of C-reactive protein by 8% and 14%, respectively, and decreased fasting insulin levels by 7% and 3%, respectively [28]. Furthermore, LPA has been positively correlated with a perception of good health as indicated by self-reports [29]. This is consistent with the negative association between LPA and stress observed in our study. A meta-analysis performed by Xie et al. (2022) showed that more than 60 min/day of LPA, measured by accelerometer, reduced the risk of cardiovascular disease (CVD) by 10% and the risk of cardiovascular death by 41% [30].

Moreover, a prospective study based on accelerometry measurements reported that mortality risk by any cause is reduced by accumulating either 375 min/day of low-intensity LPA or 325 min/day of high-intensity LPA [31]. In agreement with all these data, the participants in our study attained the LPA levels required to improve the markers of cardiometabolic health. Nevertheless, the benefits for teachers may be limited as they did not accumulate sufficient LPA.

As measured by a questionnaire, daily MPA decreased in adults during the contingency when compared with pre-contingency values [32]. The same effect was observed regarding the total daily PA (TPA) in adults as measured by questionnaires [33] or by a cell phone application [34]. The effects of the contingency were similar to those observed in this study, since during the contingency the teachers performed less daily MPA and TPA than after the lifting of the lockdown.

Some studies have shown that MVPA did not change before or during the COVID-19 lockdown, as measured by questionnaire [35] or by accelerometer [34]. Interestingly, 82% and 85% of all adults met the recommended 150 min/week of MVPA before and during the pandemic, respectively [36]. In contrast to our results, previous studies suggested that the COVID-19 lockdown did not cause physical inactivity of adults. The differences may be partially explained by the fact that the job status of the participants is unknown in some studies [36].

When teaching activities were exclusively online, 45.4% of all teachers met the WHO recommendations by performing 150 min/week of MVPA [37], whereas 54.5% of all teachers met such recommendation once the lockdown was lifted. It is well known that regular MVPA benefits health as it contributes to the prevention, treatment, and control of chronic non-communicable diseases such as CVD, hypertension, and type 2 diabetes (T2D). Regular MVPA also reduces the risk of developing metabolic syndrome, improves cognitive health and sleep, and reduces anxiety and depression symptoms [37,38].

It is encouraging that the percentage of physically active teachers increased during their on-site teaching activities. However, 45.5% of them have yet to meet the global recommendations. The risk of CVD and T2D increases by 45% and 35% for physically inactive people, respectively, whereas the risk of breast and colon cancer increases by 25% and 26%, respectively [39]. Similarly, more than 7% of all death causes (including CVD) and up to 8% of all non-communicable diseases are also attributable to physical inactivity [40]. This indicates that teachers should participate more in MVPA.

Another way to measure the PA level is by counting the steps/day. The current estimations indicate that 7000–8000 steps/day are needed to comply with the established MVPA guidelines [41]. The participants of this study failed to attain such requirements in both of its phases. Nevertheless, in this study an increase of 1100 steps/day was observed right after returning to on-site teaching activities. Rowlands et al. (2021) also found, through accelerometry, that the number of steps taken by 165 adults with T2D increased after the pandemic restrictions were lifted [42]. Within the population of adults, 1000 additional steps per day reduce the risk of mortality by any cause, as well as morbidity and cerebrovascular mortality. Furthermore, an increase of the number of steps is associated with a 0.06-point decrease of the BMI over a 5-year period for adults [43,44]. This data is consistent with the association we found, since we also observed a negative association between the number of steps and BMI. Based on the cited study and on our own results, if the teachers that participated in our study maintain a higher number of steps as they did upon returning to on-site teaching, their BMI and the risk mortality by any cause will be both reduced at medium-term.

On the other hand, SBT is associated with negative consequences for health [45]. All the teachers in the first phase of this study were engaged in SBT over half of their daytime (71%, > 10 h/day) and this behavior did not change when they returned to on-site teaching activities. Similar results were observed by Barone et al. (2021) [35]. They reported that office workers did not modify their SBT during workdays before and during the COVID-19 lockdown [35].

Most studies that reported SBT assessments before and during lockdown were based on surveys and accelerometer outcomes. All of these reported a significantly increased SBT for adults one year after the COVID-19 lockdown was established [32,33]. The difference between the results described above and those obtained in our study may be explained by (1) the moment at which SBT was evaluated as other studies obtained their results at least one year after the lockdown was established, whereas our data from the first phase was acquired approximately 1.5 years after the lockdown, when the level of restriction decreased, (2) the nature of the participants’ jobs as these were not disclosed in other studies, and (3) the instrument used to evaluate the SBT.

In line with Quinn et al. (2019) [46], during on-site classes, teachers primarily engaged in LPA and MPA and consistently took ABs; however, their SBT did not change. It is possible that, with the return to on-site activities, SBT was accumulated through other behaviors, such as passive transportation.

Adults that spent SBT > 10 h/day (as measured by accelerometry) exhibit higher fasting insulin levels, a higher prevalence of glucose intolerance, T2D, and a they are at higher risk of cerebrovascular accident when compared with other adults that accumulate SBT ˂ 6 h/day [47]. Health risks caused by SBT may be counteracted by increasing PA. Ekelund et al. (2019) [48] reported that individuals engaging in at least 1 h/day of MPA are at a lower risk of mortality caused by CVD and cancer. Notably, when individuals exceeded 1 h/day of MPA, no significant association was found between SBT and CVD-related mortality [48]. In adults between 30 and 64 years old, LPA and MVPA, measured either by questionnaire or an accelerometer device, negatively correlate with BMI and/or WC. In this scenario, a lower risk of both myocardial infarction and mortality by any cause are favored as long as they accumulate less than 6 h/day of SBT [49,50]. Our study also observed a negative association between MVPA and BMI, although teachers accumulated SBT > 10 h/day. We also detected a positive association between SBT and WC, which is consistent with another study that correlated a greater WC with an SBT value of 9.4 ± 2.3 h (as measured with accelerometry) in adults [51]. Therefore, if prolonged sedentary behavior persists alongside low engagement in moderate physical activity (less than 1 h/day), teachers in this study may be exposed to a higher risk of developing adverse health conditions over time.

SBT was positively associated with WC but not with BMI. This may be explained by the fact that sedentary behavior simultaneously promotes fat accumulation in visceral adipose tissue and the loss of muscle mass [52]. These changes in body composition may lead to an increase in WC without a corresponding increase in body weight.

Besides being physically active, another way to counteract SBT is by performing AB bouts, as disrupting SBT may improve health status. In particular, performing AB bouts ≥ 3 min (e.g., standing, walking, or stretching) every 30 min of SBT has been associated with improved outcomes regarding postprandial glucose, fasting glucose and insulin, and lipid profile, as well as the markers of inflammation and adiposity [53]. In this study, when university teachers resumed on-site teaching, they increased the number of AB bouts/day when compared with online activities during lockdown. It may be inferred that the ABs attenuate the negative effects of long periods of SBT. However, so far, no study has evaluated the AB bouts before, during, and after the COVID-19 lockdown.

### 4.2. Depression, Anxiety, and Stress

A few studies have reported the psychological status of university teachers before, during, and after the COVID-19 lockdown. Ozamiz-Etxebarria et al. (2020) [54] observed that 32.2%, 49.5%, and 49.5% of all teachers exhibited depression, anxiety, and stress symptoms, respectively, after they returned to normal on-site teaching. These percentages were higher than those reported during the sanitary contingency, when 27.5%, 26.9%, and 26.5% suffered depression, anxiety, and stress, respectively [54]. In our study, it was observed that 8.1%, 21.6%, and 21.6% of all teachers exhibited depression, anxiety, and stress symptoms, respectively, when they resumed their regular on-site teaching activities. These percentages were lower when compared with those observed during the pandemic and they even showed some improvements regarding the classification of symptoms. The results of our study may differ because of the specific moment when the DASS-21 questionnaire was applied. In the study conducted by Ozamiz, the DASS-21 was applied when the school year began in September 2020 [54]. At this moment, the return to teaching in classrooms surpassed the “new normality” conditions and, because of the increased uncertainty for teachers, they maintained or increased the depression, anxiety, and stress symptoms. In our study, the DASS-21 questionnaire was applied in April 2022, when restrictive measures were no longer applied in public spaces nor for social interactions.

This study showed a negative association between LPA and stress. When the teachers increased their LPA, the percentage of those showing stress symptoms decreased. Furthermore, stress was positively correlated with depression and anxiety, as reflected by the improvement of the respective symptoms exhibited by teachers. These results are consistent with previous reports that show a decrease of anxiety, depression, and stress symptoms caused by PA, particularly LPA [37,55].

LPA and MVPA stimulate the release of neurotransmitters such as serotonin, dopamine, and norepinephrine, which are involved in mood regulation and the reduction of perceived stress. Additionally, they help reduce cortisol levels, a key hormone associated with the stress response [56]. Moreover, previous studies have shown that light-to-moderate PA during leisure time has a stronger positive effect on depressive symptoms than work-related PA, likely due to the joy and personal preference associated with recreational activities. After the COVID-19 pandemic, people resumed activities that promote recreation, exercise, and social interaction. In our study, teachers significantly increased their LPA and MPA post-pandemic. Although the specific context of these activities was not assessed, this may partially explain the observed reduction in depressive symptoms [57].

Although energy consumption remained unchanged after returning to on-site teaching, the significant changes in macronutrient intake limit the ability to attribute body composition changes solely to PA.

The findings of this study suggest that promoting increased PA among university teachers may help reduce SBT and potentially lessen related physical and mental health risks. Therefore, it is essential to develop strategies that promote improvements in movement patterns among this population.

This study is limited by four factors. First, a small, convenient sample size was used, and consequently our results may not represent the population of all university teachers and they should be interpreted with caution. Second, it was not possible to analyze the relationship between the increased PA and ABs using cardiometabolic risk markers as there was no access to the institutional laboratories during the first phase of the study. Third, although the participants were teachers from different cities of the state of Guanajuato and from different universities, our results do not represent all university teachers and they should be interpreted with caution. Fourth, the study lacked pre-pandemic baseline data for all variables, which limits the ability to fully assess changes attributable to the lockdown period.

Despite these limitations, our findings improve the understanding of post-lockdown mobility patterns among university teachers, highlighting the role of PA in supporting mental and physical health. These results may guide future preventive and therapeutic strategies. Additional research is needed to examine movement behaviors at different educational levels and develop targeted interventions to enhance the well-being of academic populations, particularly in the context of current transformations in the university environment, such as digitalization, hyperconnectivity, and hybrid modalities.

Furthermore, it is essential to assess the impact of the restrictive actions that were a consequence of the COVID-19 pandemic on PA, especially among university faculty, who represent a cornerstone of society yet are frequently overlooked in the design of healthcare strategies. Moreover, the use of accelerometry ensures more suitable measurements of the human movement. These aspects represent the strength of our study.

## 5. Conclusions

After the COVID-19 lockdown, upon resuming on-site teaching, university teachers increased their physical activity levels, particularly LPA and MVPA, as well as their daily step count. A greater proportion of participants met the global PA recommendations during this post-lockdown period. Increases in LPA, MVPA, and number of steps were associated with improved body composition. Higher LPA levels correlated with reduced stress, and overall psychological symptoms declined. Although the number of AB bouts per day rose, teachers still accumulated over 10 h/day of sedentary behavior, a persistently high level that remains a concern and warrants further attention in future interventions.

After the COVID-19 lockdown, upon resuming on-site teaching, university teachers increased their physical activity levels, particularly LPA and MVPA, as well as their daily step count. A greater proportion of participants met the global PA recommendations during this post-lockdown period. Increases in LPA, MVPA, and number of steps were associated with improved body composition. Higher LPA levels correlated with reduced stress, and overall psychological symptoms declined. Although the number of AB bouts per day rose and several movement-related and psychological indicators improved, teachers continued to accumulate more than 10 h per day of sedentary behavior—a persistently high level that remains a concern and warrants further attention in future interventions.

## Figures and Tables

**Table 1:** Teacher characteristics during the first and second phases of the study.

Features	First Phase (n = 37) Mean ± SD or n (%)	Second Phase (n = 37) Mean ± SD or n (%)	*p*
Male	19 (51.4)	19 (51.4)	N/A
Female	18 (48.6)	18 (48.6)	N/A
Age (years)	46.27 ± 7.0	47.27 ± 7.1	N/A
Weight (kg)	73.95 ± 13.1	74.35 ± 14.5	0.62 ^a^
Body Mass Index (kg/m^2^)	27.20 ± 4.1	27.41 ± 4.8	0.50 ^a^
Normal weight Overweight Obesity	8 (22.2) 22 (59.5) 7 (18.9)	11 (29.7) 19 (51.4) 7 (18.9)	0.001 ^b^
Waist circumference (cm)	92.42 ± 11.45	88.18 ± 12.35	0.0001 ^a^
Energy consumption (kcal/día)	1762.08 ± 481.4	1881.8 ± 476.9	0.62 ^a^
Carbohydrates (%)	48.43 ± 7.98	40.07 ± 14.63	0.006 ^a^
Proteins (%)	18.25 ± 3.97	21.45 ± 7.74	0.031 ^a^
Lipids (%)	33.30 ± 7.17	38.67 ± 9.35	0.007 ^a^
Days wearing the accelerometer	n = 45	n = 37	
7 days	30 (66.6)	23 (62.16)	N/A
6 days	14 (31.1)	11 (29.7)	N/A
5 days	1 (2.2)	2 (5.40)	N/A
4 days		1 (2.7)	N/A
Average time wearing the accelerometer (min/day)	854.0 (719.0–1140.8)	875.64 (653.0–1366.0)	N/A

^a^ Paired-Samples t test. ^b^ Pearson chi-square (χ2). Not applicable, N/A.

**Table 2:** Comparison of depression, anxiety, and stress symptoms levels in teachers during the first and second phases of the study.

Psychological Aspects	First Phase (n = 37) n (%)	Second Phase (n = 37) n (%)	*p* ^b^
Depression			
Normal	29 (78.3)	34 (91.9)	0.0001
Mild	1 (2.7)	2 (5.4)	
Moderate	5 (13.5)	(0)	
Severe	0 (0)	1 (2.7)	
Extremely severe	2 (5.6)	0 (0)	
Anxiety			
Normal	31 (83.3)	29 (78.4)	0.0001
Mild	0 (0)	4 (10.8)	
Moderate	4 (10.8)	3 (8.1)	
Severe	0 (0)	0 (0)	
Extremely severe	2 (5.4)	1 (2.7)	
Stress			
Normal	28 (75)	29 (78.4)	0.0001
Mild	2 (5.4)	4 (10.8)	
Moderate	4 (10.8)	1 (2.7)	
Severe	3 (8.1)	2 (5.4)	
Extremely severe	0 (0)	1 (2.7)	

^b^ Pearson chi-square (χ2).

**Table 3:** Correlation between physical activity or sedentary behavior time with the DASS-21 variables and the anthropometric variables (n = 37).

Variable	Depression (r)	Anxiety (r)	Stress (r)	BMI (kg/m^2^) (r)	WC (cm) (r)
**Steps** per day	0.153	0.027	−0.055	−0.497 **	−0.411 *
95% CI	[−0.206, 0.457]	[−0.286, 0.339]	[−0.336, 0.302]	[−0.696, −0.225]	[−0.648, −0.094]
**Light PA** (min/day)	−0.074	−0.016	−0.284	0.163	0.062
95% CI	[−0.389, 0.222]	[−0.357, 0.362]	[−0.574, 0.090]	[−0.115, 0.450]	[−0.254, 0.408]
**Moderate PA** (min/day)	0.174	−0.073	−0.006	−0.474 **	−0.278
95% CI	[−0.174, 0.302]	[−0.416, 0.256]	[−0.323, 0.311]	[−0.689, −0.225]	[−0.589, 0.084]
**Vigorous PA** (min/day)	0.411	0.126	0.304	−0.418 **	−0.325 *
95% CI	[0.084, 0.669]	[−0.227, 0.451]	[0.001, 0.553]	[−0.673, −0.132]	[−0.610, −0.019]
**MVPA** (min/week)	0.213	−0.035	−0.033	−0.534 **	−0.324 *
95% CI	[−0.133, 0.520]	[−0.364, 0.290]	[−0.280, 0.345]	[−0.711, −0.301]	[−0.631, 0.039]
**Total PA** (min/day)	−0.047	−0.071	−0.249	−0.026	−0.081
95% CI	[−0.376, 0.267]	[−0.395, 0.295]	[−0.549, 0.094]	[−0.327, 0.296]	[−0.393, 0.253]
**SBT (min/day)**	0.187	0.119	0.022	0.163	0.300 *
95% CI	[−0.137, 0.461]	[−0.211, 0.445]	[−0.315, 0.416]	[−0.133, 0.480]	[−0.043, 0.581]
**SBT ≥ 30 min** (periods/day)	0.163	0.105	0.096	−0.010	0.019
95% CI	[−0.286, 0.353]	[−0.322, 0.357]	[−0.259, 0.458]	[−0.303, 0.383]	[−0.275, 0.396]
**ABs ≥ 3 min** (bouts/day)	0.280	−0.014	−0.001	−0.280	−0.132
95% CI	[−0.278, 0.384]	[−0.384, 0.283]	[−0.426, 0.336]	[−0.243, 0.358]	[−0.244, 0.395]
**Depression** (points)	1.000	0.484 **	0.511 **	−0.176	−0.111
95% CI	[1.000, 1.000]	[0.181, 0.713]	[0.177, 0.756]	[−0.520, 0.234]	[−0.413, 0.269]
**Anxiety** (points)	0.488 **	1.000	0.660 **	−0.036	0.012
95% CI	[0.181, 0.713]	[1.000, 1.000]	[0.375, 0.822]	[−0.377, 0.320]	[−0.306, 0.332]
**Stress** (points)	0.511 **	0.660 **	1.000	−0.087	0.043
95% CI	[0.177, 0.756]	[0.375, 0.822]	[1.000, 1.000]	[−0.420, 0.271]	[−0.284, 0.374]

Correlations were calculated using the Pearson or Spearman test. ABs, active breaks; BMI, body mass index; MVPA, moderate–vigorous physical activity; PA, physical activity; SBT, sedentary behavior time; WC, waist circumference * *p* < 0.05, ** *p* < 0.01; 95% CI: 95% confidence interval.

**Table 4:** Simple linear regression between PA and SBT with stress and anthropometric variables in second phase of the study (n = 37).

Dependent Variable	Independent Variable	β	*p*
Stress	LPA (min/day)	−0.027	**0.03**
BMI (kg/m^2^)	MVPA (min/day)	−0.116	**0.01**
BMI (kg/m^2^)	Steps per day	−0.0007	**0.02**
Waist circumference (cm)	Steps per day	−0.002	**0.04**
Waist circumference (cm)	SBT (min/día)	0.0351	**0.05**

BMI, body mass index; LPA, light physical activity; MVPA, moderate–vigorous physical activity; SBT, sedentary behavior time.

## Data Availability

The data presented in this study are available on request from the corresponding author due to ethical reasons.

## Abbreviations

The following abbreviations are used in this manuscript:

PA	Physical activity
SBT	Sedentary behavior time
ABs	Active breaks
WC	Waist circumference
LPA	Light physical activity
MPA	Moderate physical activity
VPA	Vigorous physical activity
MVPA	Moderate and vigorous physical activity
BMI	Body mass index
COVID-19	Coronavirus disease 2019
WHO	World Health Organization
CVD	Cardiovascular disease
T2D	Type 2 diabetes
